# The legume-rhizobia symbiosis can be supported on Mars soil simulants

**DOI:** 10.1371/journal.pone.0259957

**Published:** 2021-12-08

**Authors:** Randall Rainwater, Arijit Mukherjee

**Affiliations:** Department of Biology, University of Central Arkansas, Conway, AR, United States of America; Universidade de Coimbra, PORTUGAL

## Abstract

Legumes (soybeans, peas, lentils, etc.) play important roles in agriculture on Earth because of their food value and their ability to form a mutualistic beneficial association with rhizobia bacteria. In this association, the host plant benefits from atmospheric nitrogen fixation by rhizobia. The presence of nitrogen in the Mars atmosphere offers the possibility to take advantage of this important plant-microbe association. While some studies have shown that Mars soil simulants can support plant growth, none have investigated if these soils can support the legume-rhizobia symbiosis. In this study, we investigated the establishment of the legume-rhizobia symbiosis on different Mars soil simulants (different grades of the Mojave Mars Simulant (MMS)-1: Coarse, Fine, Unsorted, Superfine, and the MMS-2 simulant). We used the model legume, *Medicago truncatula*, and its symbiotic partners, *Sinorhizobium meliloti* and *Sinorhizobium medicae*, in these experiments. Our results show that root nodules could develop on *M*. *truncatula* roots when grown on these Mars soil simulants and were comparable to those formed on plants that were grown on sand. We also detected *nif*H (a reporter gene for nitrogen fixation) expression inside these nodules. Our results indicate that the different Mars soil simulants used in this study can support legume-rhizobia symbiosis. While the average number of lateral roots and nodule numbers were comparable on plants grown on the different soil simulants, total plant mass was higher in plants grown on MMS-2 soil than on MMS-1 soil and its variants. Our results imply that the chemical composition of the simulants is more critical than their grain size for plant mass. Based on these results, we recommend that the MMS-2 Superfine soil simulant is a better fit than the MMS-1 soil and it’s variants for future studies. Our findings can serve as an excellent resource for future studies investigating beneficial plant-microbe associations for sustainable agriculture on Mars.

## Background

It seems the potential next step of humanity is the travel to and inhabitation of Mars. As space travel becomes more advanced, from telescopes to orbiters and even to landers, the continuous collection of real-time data can be extradited from Mars to research labs where we learn vastly about what life on Mars might entail. When we finally touch down on Mars, the capability to grow crops sustainably will be a necessity. Martian explorations have improved our understanding of soil composition on the red planet [1–5]. For instance, while the Martian soil is known to contain a majority of essential macro-and micro-nutrients necessary to support plant growth, it also includes some potentially growth-limiting factors such as high salinity, perchlorates, sulfates, etc. [5–7]. Over the years, several soil simulants (e.g., the Johnson Space Center (JSC) Mars-1 regolith simulant, the Mojave Mars Simulant (MMS)) have been developed to simulate Martian regolith for developing future missions and conducting scientific and engineering research. The JSC Mars-1 regolith simulant was developed at the National Aeronautics and Space Administration’s (NASA) Johnson Space Center in 1997 based on the findings from the Viking and Pathfinder missions [8]. In 2007, NASA and Jet Propulsion Laboratory (JPL) scientists working on the Mars Phoenix mission developed the Mojave Mars Simulant (MMS) soils [9]. There are currently two simulants (MMS-1 and MMS-2) commercially available through The Martian Garden (Austin, TX). The MMS-1 soils use iron-rich basalt from the same deposits used by the JPL. The MMS-2 soil is a derivative of the MMS-1 soil with additives such as Iron Oxide, Magnesium Oxide, Sulfates, and Silicates, making it more than 90% similar to the Mars surface (https://www.themartiangarden.com/). These soil simulants are excellent resources for conducting diverse plant biology studies.

Several studies have reported that these regolith simulants (e.g., JSC Mars-1, MMS) supported the growth of different plants (e.g., tomato, wheat, lettuce) with varying degrees of success [10–13]. For instance, one study reported that plant growth was possible on JSC Mars-1A regolith simulant without any addition of nutrients [10]. In this study, plants such as tomato, wheat, cress, and field mustard performed particularly well. Interestingly, common vetch (a legume) did not grow on the Martian soil simulant [10]. One recent study reported that both JSC Mars-1A and MMS simulants could support the growth of *Arabidopsis thaliana* and lettuce with the addition of a nutrient supplement [13]. While additional studies still need to be conducted to optimize plant growth, these studies have shown that the Mars soil simulants have the potential to support the growth of a wide array of plants.

Besides cereal crops (rice, corn, wheat, etc.), legumes (soybeans, peas, lentils, etc.) play an important role in agriculture worldwide. For instance, legumes are an excellent source of high-quality food and feed and contribute to carbon sequestration in soils [14,15]. Legumes also facilitate the reduction of fossil energy inputs in the system due to nitrogen fertilizer reduction via their exclusive ability to form a mutualistic symbiosis with soil bacteria, rhizobia [15]. In this association, nitrogen-fixing rhizobia internally colonize the roots of legumes leading to the formation of specialized root organs called nodules. Inside these nodules, rhizobia utilize the nitrogenase enzyme to convert atmospheric nitrogen into biologically usable forms of nitrogen (ammonia) in return for carbohydrates from the host plant [16]. Due to atmospheric nitrogen fixation, legumes can grow independent of nitrogen fertilizers, a major cause of concern for the environment, the economy, and public health. Non-legume crops such as corn and rice cannot form this beneficial association with rhizobia and are therefore heavily dependent on fertilizers for their growth. Other benefits of legumes include their ability to release high-quality organic matter in the soil, facilitate nutrient cycling in the soil, soil water retention, etc. [14]. Due to these reasons, coupled with the presence of nitrogen in the Mars atmosphere, legume crops will likely be an integral part of agriculture on Mars, just like on Earth. Due to its significance in agriculture, numerous genetic studies have been conducted to investigate legume-rhizobia symbiosis. These studies have been primarily performed in model legumes such as *Medicago truncatula* and *Lotus japonicus* [16,17]. In studies involving *M*. *truncatula*, *Sinorhizobium meliloti* and *Sinorhizobium medicae* have been used as bacterial symbionts [18–20]. The last twenty years have witnessed remarkable progress in the discovery of genes and mechanisms underlying legume-rhizobia symbiosis [21]. These include identifying both plant (e.g., *DMI1*, *DMI2*, *DMI3*) and bacterial (e.g., *nif*, *nod*, *fix*) genes required for this symbiosis [16,17,21,22]. Studies have also revealed that nodules and lateral roots share some similarities in their developmental program [23–25]. However, much remains to be learned, and the future for legume biology research looks exciting. For instance, there are currently no studies investigating the legume-rhizobia symbiosis on different Mars soil simulants. The first step towards growing legumes sustainably on Mars would be to determine if this plant-microbe symbiosis can be supported on various Mars soil simulants. In the long-term, identifying the legume genes and genetic pathway(s) regulating this plant-microbe association under conditions specific to Mars will allow us to develop legumes that can adapt reasonably well to the stressful conditions on Mars.

In this study, we investigated the establishment of the legume-rhizobia symbiosis on different Mars soil simulants (different grades of the Mojave Mars Simulant (MMS)-1: Coarse, Fine, Unsorted, Superfine, and the MMS-2 simulant). We used *Medicago truncatula* as the host plant with *Sinorhizobium meliloti* and *Sinorhizobium medicae* as the symbiotic partners. We compared plant mass, lateral roots, and nodules formed on plants grown on different Mars soil simulants. We also checked for *nifH* expression inside the root nodules formed on plants grown on these different soils. Our study will be an excellent resource for future studies seeking to investigate the legume-rhizobia symbiosis on Mars soils.

## Results

### Total mass of *Medicago truncatula* plants were comparable between MMS-2 soil and sand but was lesser on MMS-1 soils than on sand

**Fig 1:**
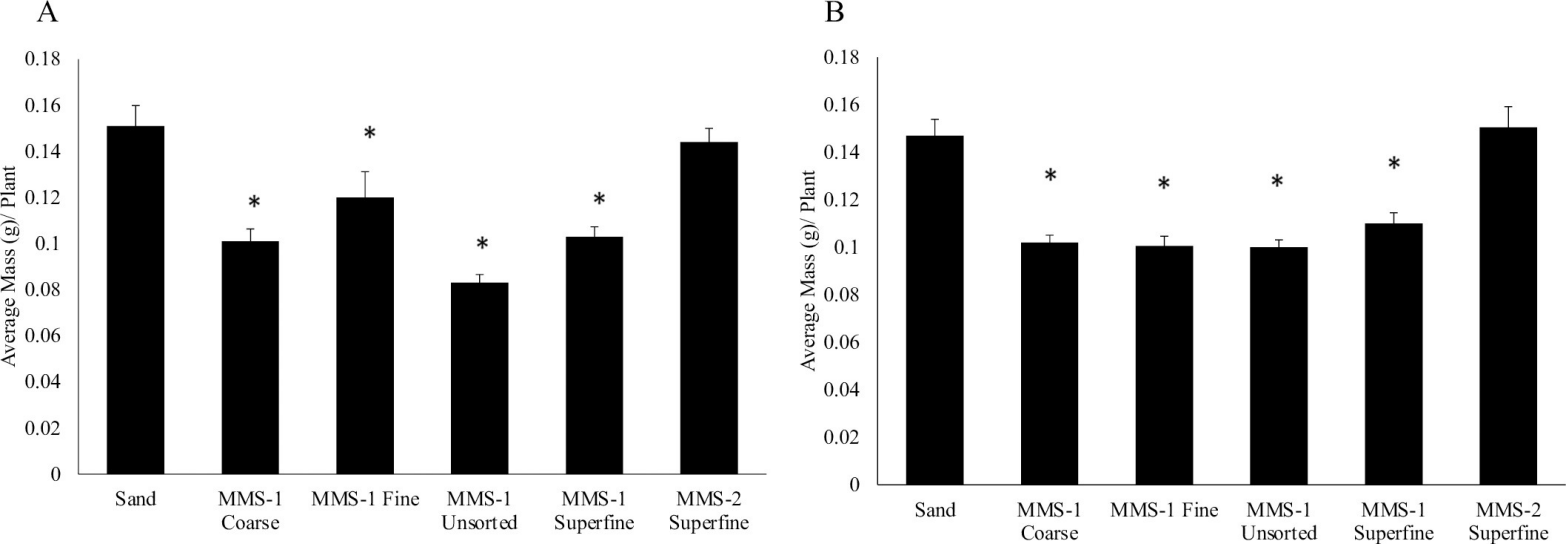
Average plant mass when grown on the different Mars soil simulants and sand. Total plant mass data was collected 14 days post inoculation (dpi) with *S*. *meliloti* **(A)** and *S*. *medicae* **(B).** Total plant mass was significantly (p<0.05) reduced in plants grown on MMS-1 Coarse, MMS-1 Fine, MMS-1 Unsorted, and MMS-1 Superfine when compared to plants grown on sand. Plants grown on MMS-2 Superfine do not show a reduction in plant mass compared to plants grown on sand. We observed a similar trend in plant mass, when the plants were inoculated with *S*. *medicae* **(B)**. Asterisk * denotes significant difference between the conditions by *t-*test (p<0.05). Data represents the average at least three experimental replications (n = 8–15) +/- SE.

We compared the total mass of wild-type (A17) *Medicago truncatula* plants grown on the different Mars soil simulants (different grades of the Mojave Mars Simulant (MMS)-1: Coarse, Fine, Unsorted, Superfine, and the MMS-2 simulant) obtained from The Martian Garden (www.themartiangarden.com), with plants grown on sand. In this study, we used sand (Quikrete all-purpose sand) as our control substrate as it’s easier to retrieve intact root nodules from sand than from soil, and *M*. *truncatula* grows well on sand [26,27]. Total plant mass was measured 14- days post inoculation (dpi) with *Sinorhizobium meliloti* (Fig 1A) or *Sinorhizobium medicae* (Fig 1B). When inoculated with *S*. *meliloti*, our results show that total mass decreased for plants grown on MMS-1 Coarse (by 1.5-fold), MMS-1 Fine (by 1.26-fold), MMS-1 Unsorted (by 1.82-fold), and MMS-1 Superfine (by 1.47-fold) soils when compared to plants grown on sand (Fig 1A). Interestingly, the total mass of plants grown on MMS-2 Superfine soil was comparable to the total mass of plants grown on sand (Fig 1A). When inoculated with *S*. *medicae*, we observed a similar trend in plant mass. For instance, total mass decreased for plants grown on MMS-1 Coarse (by 1.44-fold), MMS-1 Fine (by 1.46-fold), MMS-1 Unsorted (by 1.47-fold), and MMS-1 Superfine (by 1.34-fold) soils when compared to plants grown on sand (Fig 1B). We also observed that there was no significant difference in total mass between the plants grown on MMS-2 Superfine soil and the plants grown on sand (Fig 1B).

### The average lateral root number was decreased on plants grown on the different Martian soil simulants than on sand

**Fig 2:**
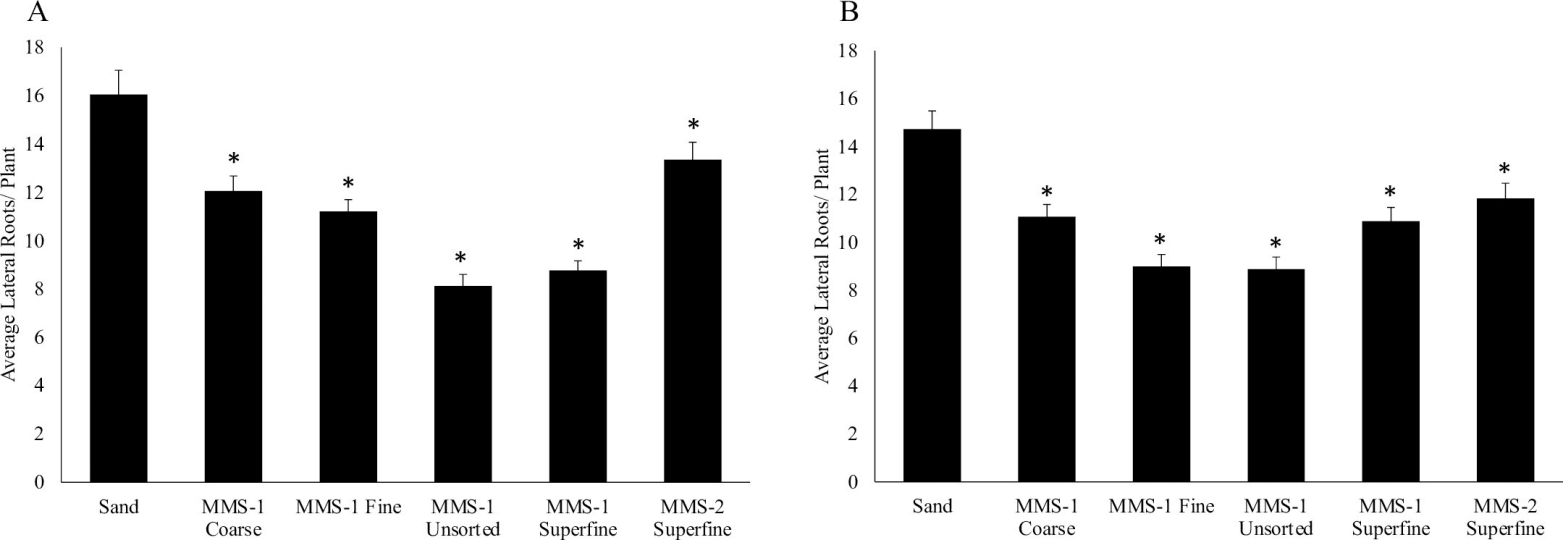
Average lateral roots on plants grown on the different Mars soil simulants and sand. Total plant lateral root data was collected 14 days post inoculation with *S*. *meliloti* **(A)** and *S*. *medicae* **(B).** Average number of lateral roots decreased significantly (p<0.05) in plants grown on all Mars soil simulants (MMS-1 Coarse, MMS-1 Fine, MMS-1 Unsorted, MMS-1 Superfine, and MMS-2 Superfine) when inoculated with *S*. *meliloti*. **(A)**. We observed a similar pattern in significant reduction of average lateral roots/plant when inoculated with *S*. *medicae* **(B)**. Asterisk * denotes significant difference between the conditions by *t*-test (p<0.05). Data represents the average at least three experimental replications (n = 9–15) +/- SE.

Next, we measured the average number of lateral roots formed per plant when grown on the different Mars soil simulants and compared to average lateral root numbers on plants grown on sand. We counted average lateral root numbers 14- dpi with *S*. *meliloti* (Fig 2A) or *S*. *medicae* (Fig 2B). When inoculated with *S*. *meliloti*, our results show that the average lateral root number was reduced for plants grown on MMS-1 Coarse (by 1.33-fold), MMS-1 Fine (by 1.43-fold), MMS-1 Unsorted (by 2.00-fold), MMS-1 Superfine (by 1.83-fold), and MMS-2 Superfine (by 1.20-fold) soils when compared to plants grown on sand (Fig 2A). When inoculated with *S*. *medicae*, we observed a similar trend in lateral root number per plant. For instance, average lateral root number was reduced for plants grown on MMS-1 Coarse (by 1.52-fold), MMS-1 Fine (by 1.86-fold), MMS-1 Unsorted (by 1.88-fold), MMS-1 Superfine (by 1.54-fold), and MMS-2 Superfine (by 1.41-fold) soils when compared to plants grown on sand (Fig 2B). These results indicate that while lateral roots can be formed on plants grown on the different Martian soil simulants, average lateral root number was significantly reduced on plants grown on these soils if compared to plants grown on sand (Fig 2A and 2B).

### The average number of nodules formed on *Medicago truncatula* roots was comparable when grown on the different Martian soil simulants and sand

**Fig 3:**
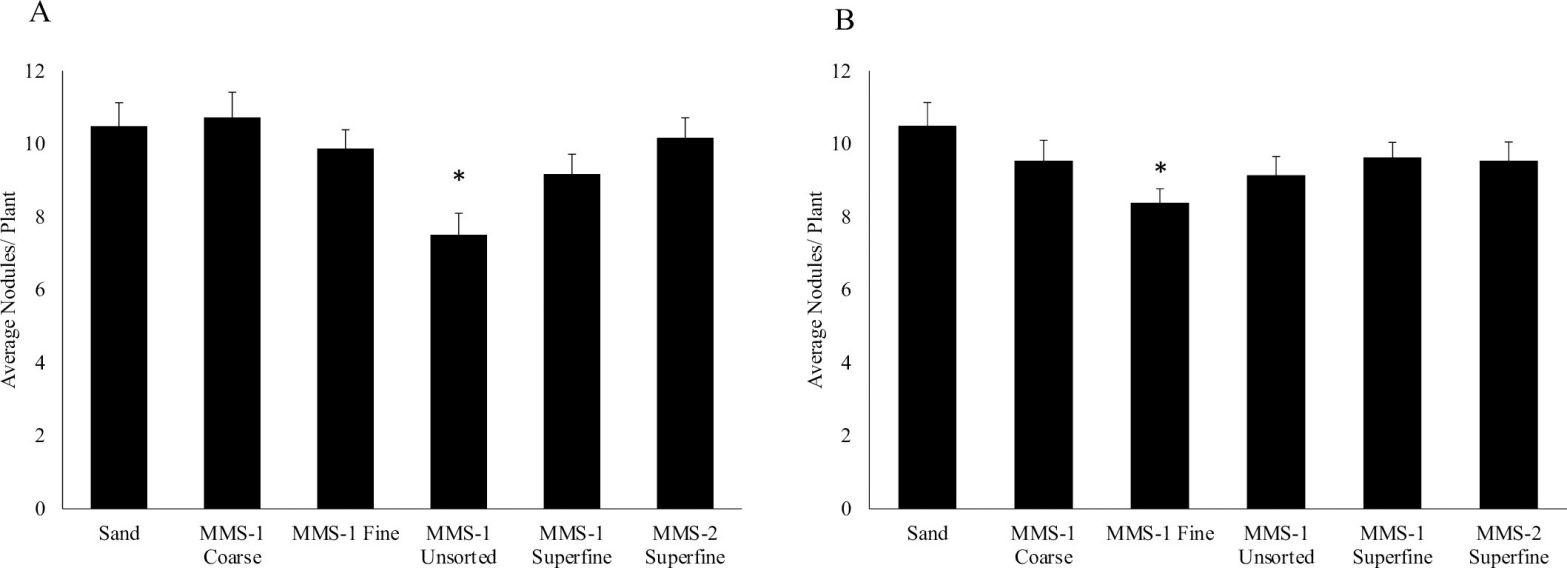
Average root nodules on plants grown on the different Mars soil simulants and sand. Total plant root nodule data was collected 14 days post inoculation with *S*. *meliloti* **(A)** and *S*. *medicae* **(B).** Plants grown on MMS-1 coarse, MMS-1 fine, MMS-1 superfine, and MMS-2 superfine soils showed similar average nodule number compared to average nodule number on plants grown on sand, when inoculated with *S*. *meliloti* **(A).** However, plants grown on MMS-1 Unsorted soil showed significant (p<0.05) decrease in average nodule number, when inoculated with *S*. *meliloti*, compared to sand **(A).** Plants grown on MMS-1 coarse, MMS-1 unsorted, MMS-1 superfine, and MMS-2 superfine soils showed similar average nodule number compared to average nodule number on plants grown on sand, when inoculated with *S*. *medicae* **(B)**. However, plants grown on MMS-1 fine soil showed significant (p<0.05) decrease in average nodule number, when inoculated with *S*. *medicae*, compared to sand **(B)**. Asterisk * denotes significant difference between the conditions by *t*-test (p<0.05). Data represents the average at least three experimental replications (n = 9–15) +/- SE.

We were interested to determine if root nodule formation was affected on *M*. *truncatula* plants grown on the different Mars soil simulants. We measured average nodule numbers formed per plant 14-dpi with *S*. *meliloti* (Fig 3A) or *S*. *medicae* (Fig 3B). When inoculated with *S*. *meliloti*, our results show that the average nodule number was similar for plants grown on MMS-1 Coarse, MMS-1 Fine, MMS-1 Superfine, MMS-2 Superfine, and sand (Fig 3A). However, the average nodule number decreased for plants grown on MMS-1 Unsorted (by 1.40-fold) soil when compared to plants grown on sand (Fig 3A). When inoculated with *S*. *medicae*, the average nodule number was similar for plants grown on MMS-1 Coarse, MMS-1 Unsorted, MMS-1 Superfine, MMS-2 Superfine and sand (Fig 3B). But the average nodule number decreased for plants grown on MMS-1 fine (by 1.41-fold) when compared to plants grown on sand (Fig 3B). We also observed that the average number of nodules formed per plant on the different soils did not change much with the strain of bacteria used in these experiments (Fig 3).

### *nif*H expression was detected on nodules formed on plants grown on the different Mars soil simulants

**Fig 4:**
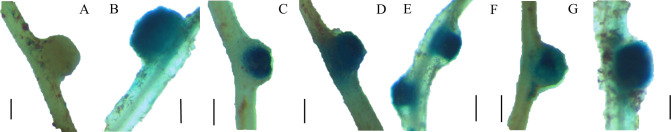
*nif*H expression in the root nodules of plants grown on the different Mars soil simulants and sand. *M*. *truncatula* plants were inoculated with *S*. *meliloti* 1021 carrying the P*nif*H::*uidA* fusion revealing *nif*H expression inside the nodules, 14 days post-inoculation. (**A**) represents an unstained root nodule, (**B**-**G**) represent *nif*H expressing root nodules from *M*. *truncatula* plants when grown on sand (**B**), MMS-1 Coarse (**C**), MMS-1 Fine (**D**), MMS-1 Unsorted (**E**), MMS-1 Superfine (**F**), and MMS-2 Superfine (**G**). Bar = 0.5 mm.

Finally, we determined the expression of the bacterial *nif*H gene inside the nodules formed on *M*. *truncatula* plant roots. The *nif*H gene encodes the homo-dimeric iron protein of the nitrogenase enzyme complex [28]. It is a reliable reporter gene for the nitrogen fixation pathway and has been utilized in several studies [20,29,30]. To determine whether the *nif*H gene was expressed in the root nodules, we inoculated the *M*. *truncatula* plants growing on the different soils with an *S*. *meliloti* 1021 strain carrying the *nif*H promoter-GUS reporter gene fusion [20]. Our results show that the *nif*H promoter was active in the nodules of *M*. *truncatula* plants grown on all the soils, 14-dpi (Fig 4).

## Discussion

Legumes are an integral part of agriculture on Earth due to several factors, including their immense contribution to sustainable agriculture [14,15]. This is mainly possible because of their ability to benefit from atmospheric nitrogen fixation by forming beneficial alliances with soil bacteria, rhizobia. The presence of nitrogen in the Mars atmosphere (around 2.7%) and ‘fixed’ nitrogen in Martian soil [31,32] offers the possibility to take advantage of one of the most important plant-microbe associations on Earth. Legumes can be used to enrich Martian soils with nitrogen which will be helpful for non-legume plants that are not as efficient in nitrogen uptake. While some studies have investigated the growth of different crops on Mars soil simulants, none have investigated if these soils can support the legume-rhizobia symbiosis. In this study, we used the model legume *Medicago truncatula* and its symbiotic partners, *Sinorhizobium meliloti* and *Sinorhizobium medicae* to determine if different Mars soil simulants can support this important plant-microbe association. We used different Mars soil simulants (different grades of the Mojave Mars Simulant (MMS)-1: Coarse, Fine, Unsorted, Superfine, and the MMS-2 simulant) available through The Martian Garden (Austin, TX) in these experiments. We used sand as our control substrate as *M*. *truncatula* grows well on it, and it’s easier to retrieve intact root nodules from sand than from soil [26].

First, we determined if total plant mass differed when grown on the different Mars soil simulants versus sand. Our results show that when the *M*. *truncatula* plants were grown on the MMS-1 soils, the total mass was comparatively lesser than when grown on sand. The different grades of the MMS-1 soils did not affect the results indicating that grain size is not a major factor. However, when grown on MMS-2 soil, the plants had a greater mass than when grown on the MMS-1 soils. These results suggest that the added compounds (Iron Oxide, Magnesium Oxide, Sulfates, Silicates) in the MMS-2 soil are likely playing a role. It is important to note that once planted, besides bacterial inoculation and regular watering, none of these plants were supplemented with any other nutrient solution. Another important observation is that the pattern in the results did not change with the rhizobial strain used. These results are encouraging as the MMS-2 soil has a high degree of similarity (> 90%) to the Mars surface (https://www.themartiangarden.com/). Studies have shown that root nodules and lateral roots share overlapping developmental programs, with some plant genes modulating lateral root and nodule numbers in *M*. *truncatula* [24,25]. So, we were interested in determining if lateral root formation was affected in *M*. *truncatula* plants when grown on the different MMS soils. Our results show that lateral root numbers per plant were reduced on plants grown on both MMS-1 and MMS-2 soils compared to sand. These results did not change with the strain of bacteria used for inoculation. Soil compaction can lead to the formation of fewer lateral roots [33]. Since sand is slightly denser (1.5 g/cm^3^) than the Mars soil simulants (1.12–1.37 g/cm^3^), it is expected to be more compact. Therefore, the reduction in lateral root numbers in plants grown on the Mars soil simulants is likely not due to soil compaction. Whether the chemical composition of these MMS soil simulants affects lateral root formation in *M*. *truncatula* roots will require further investigation. Next, we were interested in studying root nodule formation in the *M*. *truncatula* plants grown on the different Mars soil simulants. Our results showed that root nodules developed in the *M*. *truncatula* plants when grown on all the Mars soil simulants. Also, we observed that the average number of nodules formed on the *M*. *truncatula* roots when grown on the Mars soil simulants were mostly comparable to those formed on plants grown on sand. It was encouraging to observe that root nodule formation was not dependent on the type of simulant used in this study. The average number of root nodules was also comparable between plants inoculated with *S*. *meliloti* or *S*. *medicae*. Finally, we investigated if the *nif*H gene was expressed inside the *M*. *truncatula* root nodules. The bacterial *nif*H gene encodes a nitrogenase reductase and is required for atmospheric nitrogen fixation [28]. The P*nif*H::*uidA* fusion serves as an excellent marker for nitrogenase gene expression and is routinely used in legume-rhizobia studies [29,30]. Our results show that the *nif*H promoter was active in root nodules formed on *M*. *truncatula* plants when grown on the different Mars soil simulants. The ability of these soils to support root nodule formation and subsequent *nif*H expression inside the nodules is extremely promising for future studies on Martian soils.

Based on our findings, we can summarize that the Mars simulant soils used in this study can support the symbiotic association between *M*. *truncatula* and its rhizobial partners. We also recommend that the MMS-2 soil is a better fit than MMS-1 for further studies investigating this association. However, it will be interesting to conduct similar studies on other Martian simulants as well. For instance, the Mars Global Simulant (MGS-1) soil and its variants are excellent candidates for future studies [34]. However, similar to the simulants used in this study, the MGS-1 simulants do not include the highly hazardous components in the Martial soil that pose challenges for plant growth, such as perchlorates. It will be interesting to study if the addition of perchlorates in the soil simulants affects root nodule development. One recent study reported the development of five new regolith and bedrock simulants [35]. These simulants are chemically and mineralogically comparable to Martian regolith and can be used for future studies involving Martian soil as a plant growth medium. We acknowledge that our studies were performed on trays and not in full soil cultivation, which might be the case on Mars. We also recognize that the atmospheric conditions for the experiments in this study do not reflect the conditions on Mars. For instance, the lower amount of nitrogen in the Mars atmosphere than Earth may affect the legumes’ ability to fix atmospheric nitrogen. Future studies can investigate legume-rhizobia symbiosis in atmospheric conditions closer to Mars. However, in all likelihood, all plants will be grown on Mars under greenhouse conditions with an enhanced atmosphere, supplemental lighting, and moderated temperatures. Overall, our findings can serve as an excellent resource for future studies investigating beneficial plant-microbe associations on Mars soil simulants.

## Materials and methods

### Plant preparation and growth conditions

We utilized wild-type *Medicago truncatula* Jemalong A17 seeds for all experiments in this study. The seeds were surface-sterilized in concentrated sulfuric acid (1M) for 8 minutes, then washed four times with sterile diH_2_O followed by one rinse with 5.6% (v/v) sodium hypochlorite solution. The seeds were rinsed an additional three times with sterile diH_2_O and allowed to imbibe in sterile diH_2_O for a minimum of 2 h in a sterile environment. After the imbibition period, sterilized seeds were transferred to 9-cm Petri plates (#633185, Greiner Bio-one, Monroe, NC, USA) containing 1% (w/v) plant agar (#A038-2.5KG, Caisson Laboratories Inc., Smithfield, UT, USA) supplemented with 1 mM Gibberellic acid (GA3) medium under sterile conditions. The plates were sealed with parafilm, wrapped completely in aluminum foil, and placed at 4°C for a minimum of 48 h, removed from the refrigerator, and then placed at room temperature (25°C) for an additional 48–96 h to allow the seeds to germinate. The germinated seedlings were planted on trays with inserts (2.35” X 1.94” X 2.23”; CN-IKN, Greenhouse Megastore, USA) filled with the different Mars soil simulants. These plants were allowed to grow in a temperature-controlled (22°C—25°C) greenhouse with a 16-hour photoperiod. The plants were watered every 24–48 hours with each plant receiving 1ml of water. The plants were grown for seven days before inoculation with bacteria. For this study, we used different Mars soil simulants (different grades of the Mojave Mars Simulant (MMS)-1: Coarse, Fine, Unsorted, Superfine, and the MMS-2 simulant) available through The Martian Garden (Austin, TX) along with sand. We used sand (Quikrete all-purpose sand) as our control substrate in this study. In this study, all experiments were replicated at least three times.

### Bacterial growth and inoculation

Rhizobial strains *Sinorhizobium meliloti* 2011, *Sinorhizobium medicae* WSM419 and *Sinorhizobium meliloti* 1021 containing the P*nif*H::*uidA* fusion were used for inoculation. The bacterial strains were grown in Tryptone-Yeast extract medium and supplemented with appropriate antibiotics at 30°C to an optical density (600 nm) of 0.8. Each plant was inoculated with the appropriate bacterium at a concentration of 1x10^8^ cells/ml. Following inoculation, the plants were grown in the greenhouse as described above. The different plant phenotypes (total plant mass, lateral root number, average nodule number) were recorded 14 days post-inoculation.

### *nif*H expression staining and visualization

For *nif*H expression, the root nodules were harvested 14 days post-inoculation with *Sinorhizobium meliloti* 1021 containing the P*nif*H::*uidA* fusion. *nifH* expression was visualized via staining for β-glucuronidase activity with 5-bromo-4-chloro-3-indolyl-D-glucuronic acid (X-gluc) as described in [36] with minor modifications. Following staining, the roots were fixed in 90% ethanol before brightfield imaging. The root nodule images were taken with a Leica MZ6 stereomicroscope (Leica Microsystems Inc., Buffalo Grove, IL, USA).

### Statistical analysis

Statistical analysis for total plant mass, average number of lateral roots, and average number of root nodules was analyzed via unpaired student’s *t*-test with an α-value of 0.05. Differences were considered statistically significant if p < 0.05.

## Supporting information

S1 Raw data (XLSX)
